# All-trans retinoic acid modulates mitogen-activated protein kinase pathway activation in human scleral fibroblasts through retinoic acid receptor beta

**Published:** 2013-08-06

**Authors:** Lijun Huo, Dongmei Cui, Xiao Yang, Zhenya Gao, Klaus Trier, Junwen Zeng

**Affiliations:** 1 State Key Laboratory of Ophthalmology,Zhongshan Ophthalmic Center, Sun Yat-sen University, Guangzhou, P. R. China; 2 The First Affiliated Hospital, Sun Yat-sen University, Guangzhou, P. R. China; 3 Trier Research Laboratories, Hellerup, Denmark

## Abstract

**Purpose:**

All-trans retinoic acid (ATRA) is known to inhibit the proliferation of human scleral fibroblasts (HSFs) and to modulate the scleral intercellular matrix composition, and may therefore serve as a mediator for controlling eye growth. Cell proliferation is regulated by the mitogen-activated protein kinase (MAPK) pathway. The aim of the current study was to investigate whether changed activation of the MAPK pathway could be involved in the response of HSFs exposed to ATRA.

**Methods:**

HSFs were cultured in Dulbecco Modified Eagle's Medium/F12 (DMEM/F12) and exposed to 1 μmol/l ATRA for 10 min, 30 min, 1 h, 8 h, or 24 h. The activation of extracellular signal-regulated kinase (ERK 1/2), p38, and c-Jun N-terminal kinase (JNK) in HSFs was assessed with western blot analysis and immunocytofluorescence.

**Results:**

After exposure to ATRA for 24 h, the HSFs appeared shrunken and thinner than the control cells. The intercellular spaces were wider, and the HSFs appeared less numerous than in the control culture. Western blot showed decreased activation of ERK 1/2 in the HSFs from 30 min (p=0.01) to 24 h (p<0.01) after the start of exposure to ATRA, and increased activation of the JNK protein from 10 to 30 min (p<0.01) after the start of exposure to ATRA. Indirect immunofluorescence confirmed changes in activation of ERK 1/2 and JNK in HSFs exposed to ATRA. No change in activation of p38 in HSFs was observed after exposure to ATRA. Pretreatment of the HSFs with LE135, an antagonist of retinoic acid receptor beta (RARβ), abolished the ATRA-induced changes inactivation of ERK 1/2 and JNK.

**Conclusions:**

ATRA inhibits HSF proliferation by a mechanism associated with modulation of ERK 1/2 and JNK activation and depends on stimulation of retinoic acid receptor beta.

## Introduction

Myopia is a common visual disorder affecting about 70% of young people living in urban area in southern China [1]. The prevalence of high myopia, which may cause sight-threatening complications, seems to be increasing in several parts of the world [2]. Scleral fibroblasts play a key role in the scleral remodeling that accompanies the excessive eye elongation that takes place during myopia progression [3-5]. All-trans retinoic acid (ATRA) is believed to act as a modulator of ocular growth [6-8]. ATRA is a potent regulator of cell proliferation and differentiation in various types of cells, including human scleral fibroblasts (HSFs). Previous studies have shown that retinoic acid receptors (RARs) and retinoid X receptors (RXRs) are present in cultured HSFs, and that ATRA inhibits the proliferation of HSFs [9,10].

The effects of retinoic acid (RA) on cells are mainly mediated by two families of nuclear receptors: RAR isotypes (α, β, and γ) and RXR isotypes (α, β, and γ) [11]. The RAR family is activated by ATRA and 9-cis retinoic acid (9CRA), while the RXR family is activated exclusively by 9CRA. The binding of a retinoic acid ligand induces a series of conformational changes, enabling the receptor to recruit histone acetyltransferases (HATs) and other complexes that activate target gene expression [12].

The exact mechanism by which ATRA inhibits the proliferation of HSFs remains unclear. We have previously found that ATRA increases the expression of RARβ, at the messenger ribonucleic acid (mRNA) and protein level (our unpublished data). The three major mitogen-activated protein kinase (MAPK) pathways, extracellular signal-regulated kinase (ERK 1/2) MAPK, p38 MAPK, and c-Jun N-terminal kinase (JNK) MAPK, appear to play important roles in regulating cell proliferation [13,14]. We therefore investigated whether the response of HSFs to ATRA was associated with changes in activation of the MAPK pathway or directly by RARβ.

LE135 is a synthetic antagonist of RARβ and displays moderate selectivity for RARβ over RARγ [15]. LE135 can inhibit ATRA-induced transcriptional activation of RARβ but not RARα or RARγ on several retinoic acid response elements (RAREs). Thus, LE135 is a valuable tool for studying RARβ function [16,17]. We also used LE135 to block the effect of RARβ on HSFs to confirm our previous finding.

## Methods

### Cell culture and cell treatment

At least three separate experiments from cell culture to western blot analysis and indirect immunofluorescence were performed in each group. This study was approved by the Ethics Committee of Zhongshan Ophthalmic Center, Sun Yat-sen University, China, and complied with the tenets of the Declaration of Helsinki for Research Involving Human Tissue. Primary HSFs were cultured and identified as previously described [18]. Briefly, scleral tissue was obtained from the Guangdong Province Eye Bank, China, and immediately washed in a phosphate buffered solution (PBS: containing KH_2_PO_4_, Na_2_HPO_4_, NaCL, KCL, pH 7.4 ) containing 1X penicillin-streptomycin solution (Invitrogen, Carlsbad, CA). The posterior sclera was trimmed into 1 mm^2^ pieces and placed in 25 cm^2^ plastic culture bottles in Dulbecco’s modified Eagle's medium (DMEM/F12; Gibco, Grand Island, NY) with 1X penicillin-streptomycin solution and 15% fetal bovine serum (FBS; Gibco). The sclera was then incubated at 37 °C in a humidified incubator containing 5% CO_2_. The medium was changed every 3 days. The cells were trypsinized and subcultured at a split ratio of 1:2, when a heavy primary monolayer was achieved. The third or fourth fibroblast passage was used for the experiment. The purity of the fibroblast cultures was confirmed, as previously described [18], by staining for vimentin and stain resistance for cytokeratin, desmin, and S-100, using indirect immunofluorescence. The HSFs were cultured in 10 cm dishes (Corning, Corning, NY) until 70%–80% confluence occurred for extracting protein for western blot, and cultured on coverslips in six-well plates (Corning) for immunofluorescence microscopy.

### Western blot analysis

All-trans retinoic acid (Sigma-Aldrich, St. Louis, MO) and LE135 (Tocris Bioscience, Bristol, UK) were dissolved in dimethyl sulfoxide (DMSO; MP Biomedicals, Santa Ana, CA) to a stock concentration of 10 mmol/l, which was further diluted to the working concentration of 1 μmol/l, with DMEM/F12, before immediate use or until frozen in aliquots at −20 °C, avoiding light. Before treatment, the HSFs were preincubated with DMEM/F12 without FBS for 24 h and treated with 1 μmol/l ATRA, or pretreated with 1 μmol/l LE135 for 24 h. The medium was then changed to 1 μmol/l ATRA for the appropriate treatment time.

HSFs were treated with 1 μmol/l ATRA for 10 min, 30 min, 1 h, 8 h, or 24 h, and then harvested. HSFs were treated with 0.1% DMSO as the control. The following procedures were conducted on ice. HSF lysate was prepared with 10XRIPA buffer (Cell Signaling Technology, Beverly, MA) diluted into 1X radioimmunoprecipitation assay (RIPA) buffer, with ddH2O, and by adding protease inhibitor cocktail tablets (Roche Applied Science, Mannheim, Germany) and Halt phosphatase inhibitor cocktail (Thermo-Scientific, Dreieich, Germany) immediately before use. After the cells were washed three times with PBS at 4 °C, 150 μl lysate was added to each dish, and then the cells were scraped and collected in a 1.5 ml microcentrifuge tube. The cell lysates were vortexed thoroughly and placed on 4 °C ice for 30 min and then subjected to centrifugation (13,200 × *g*) at 4 °C for 30 min. About 30–50 μg protein was loaded in each lane of 10% sodium dodecyl sulfate-polyacrylamide gels, transferred onto polyvinylidene difluoride membranes (Roche) for electrophoresis, and blocked in 5% w/v fat-free dry milk, 1X Tris buffered saline with Tween (TBST, 0.1% Tween-20, 150 mM NaCl, and 50 mM Tris at pH 7.5) for 1 h. The membranes were exposed to antibodies of phospho-p44/42 MAPK (phospho-ERK 1/2; Thr-202/Tyr-204; 1:5,000), p44/42 MAPK (ERK 1/2; 1:3,000), phospho-p38 MAPK (Thr180/Tyr182; 1:1,000), p38 MAPK (1:1,000), phospho-c-Jun (phospho-JNK; Ser63; 1:500), and c-Jun (JNK; 1:1,000; Cell Signaling Technology) diluted in 5% w/v bovine serum albumin (BSA) and 1X TBST, and incubated overnight at 4 °C. The membranes were washed in TBST, three times for 5 min, and then incubated with a secondary horseradish peroxidase (HRP)-labeled antibody (Cell Signaling Technology) at 1:5,000 for 1 h. Protein bands were visualized with the use of a chemiluminescence Phototope-HRP (Millipore, Billerica, MA), and exposed to a negative film, developed, and fixed. The film was scanned and then analyzed with BIO-RAD Quantity One Imaging software (Bio-Rad Laboratories, Hercules, CA). The same membranes were stripped with TBST and reanalyzed using antiglyceraldehyde-3-phosphate dehydrogenase (GAPDH) antibodies (Proteintech Group, Chicago, IL) as an internal control. The relative level of protein was expressed as the density ratio of the protein compared to GAPDH in the same sample.

### Indirect immunofluorescence

HSFs grew on coverslips until 70%–80% confluence, washed three times with 0.01 M PBS, fixed with methyl alcohol for 15 min at −20 °C, incubated with 10% normal goat serum (Boster Biologic Technology, Wuhan, China) for 30 min at 37 °C, and then incubated at 4 °C overnight with primary rabbit antibodies of phospho-p44/42 MAPK (Thr-202/Tyr-204) diluted at 1:100 in PBS, phospho-p38 MAPK (Thr180/Tyr182) diluted at 1:50 in PBS, or phospho-c-Jun (Ser63) diluted at 1:50 in PBS. Cells were incubated in PBS without primary antibodies as a negative control. The coverslips were then washed with PBS three times (each time for 5 min) and exposed to antirabbit immunoglobulinG fragment (Alexa Fluor 488-conjugate) secondary antibody (Cell Signaling Technology), diluted at 1:1,000 in PBS at 37 °C for 1 h. The coverslips were washed with PBS three times (each time for 5 min), stained with propidium iodide (Sigma Aldrich) to stain the cell nuclei, and stored in 4 °C refrigerator protected from light. Immunofluorescent images were taken with a confocal microscope (LSM 510 META, Carl Zeiss, Jena, Germany) within 24 h.

### Statistical analysis

Results were obtained from at least three separate repeated experiments from cell culture to western blot analysis and indirect immunofluorescence, and data expressed as mean±standard deviation (SD). Statistical analysis (SPSS version 12.0, SPSS, Chicago, IL) used one-way analysis of variance (ANOVA). Statistical significance was defined as p<0.05.

## Results

### Primary culture of human scleral fibroblasts and morphological changes in human scleral fibroblasts exposed to all-trans retinoic acid

**Figure 1:**
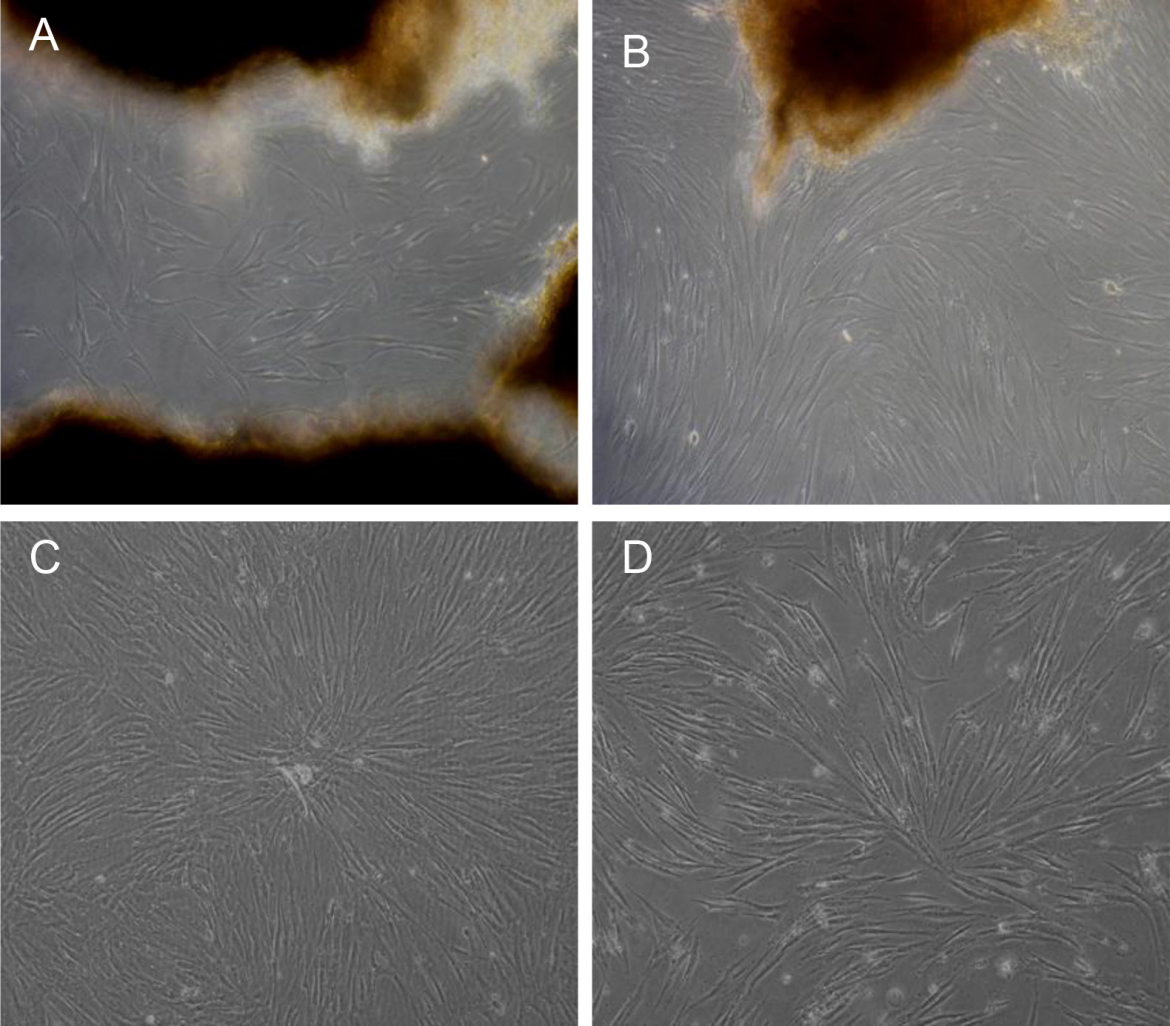
Primary tissue culture of human scleral fibroblasts (HSFs) and the morphology changes in human scleral fibroblasts treated with all-trans retinoic acid for 24 h. **A**: HSFs migrated from the sclera tissue after 2 weeks of culture. **B**: HSFs populated the surroundings and arranged in a radiating pattern for another 1 to 2 weeks. **C**: Normal group without all-trans retinoic acid (ATRA) cultured for 1 month. **D**: After being treated with 1 μmol/l ATRA for 24 h, the HSFs shrank, the apparent number of HSFs decreased, and the intercellular space widened compared with the normal group. The original magnification was 100X.

After being cultured for about 2 weeks, the HSFs started migrating from the scleral tissue and populated the surroundings, as shown in Figure 1A. The HSFs exhibited a uniform shape. The growth was compact and progressed in a radiating pattern for another week or two, as shown in Figure 1B.After being treated with 1 μmol/l ATRA for 24 h, the HSFs appeared thinner and shrunken. The apparent number of HSFs had decreased, and the intercellular spaces had widened (Figure 1C) compared with those of the control culture (Figure 1D).

### Decreased phosphorylation of extracellular signal-regulated kinase in human scleral fibroblasts exposed to all-trans retinoic acid

To evaluate the effect of ATRA on HSF proliferation, we used indirect immunofluorescence to detect the protein distribution and changes in the MAPK pathway in the HSFs. Phospho-ERK 1/2 was expressed and distributed in the HSFs’ cytoplasm and nucleus.

**Figure 2:**
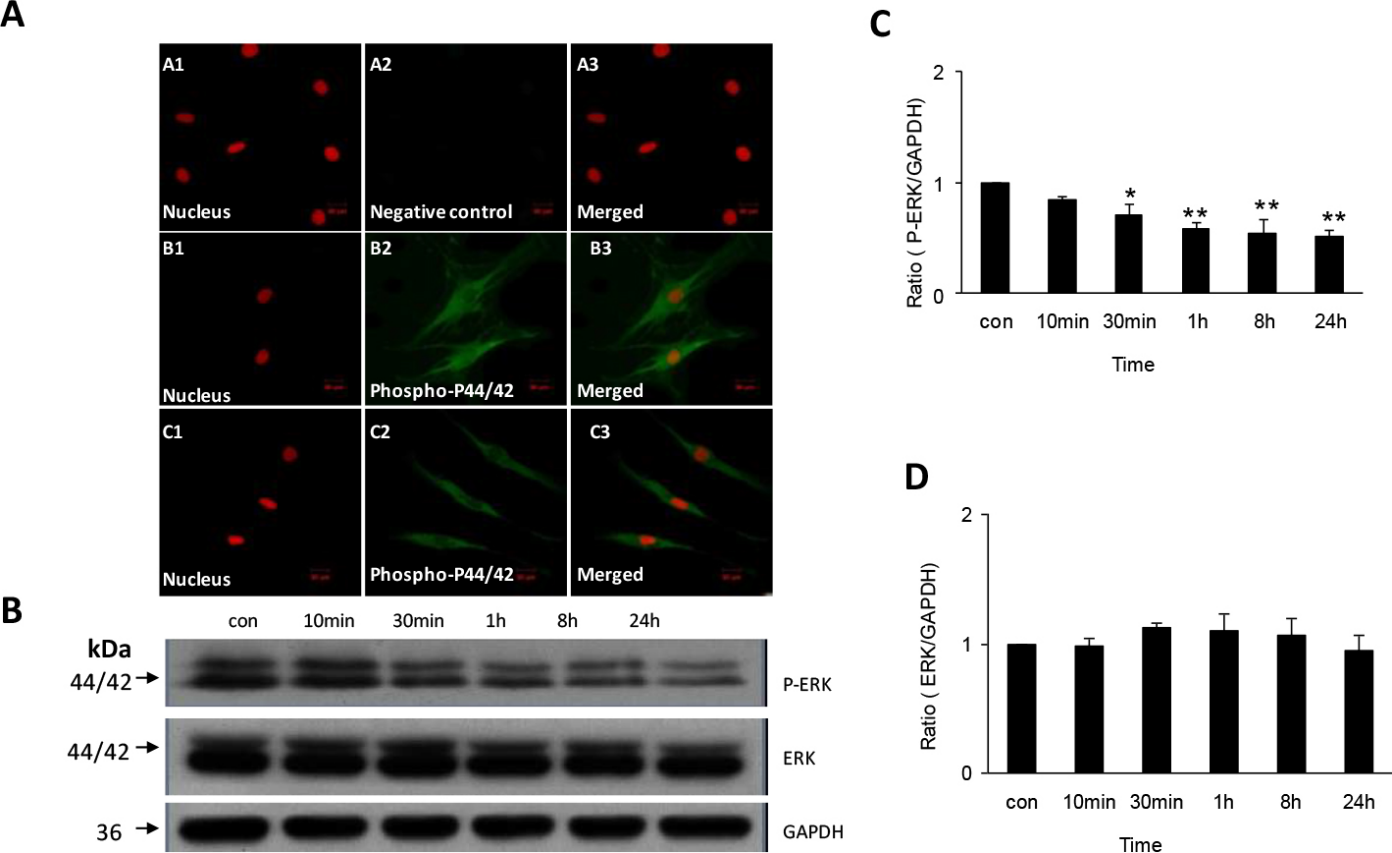
Change of expression of phospho-extracellular signal–regulated kinase and extracellular signal-regulated kinase proteins in human scleral fibroblasts treated with 1 µmol/l all-trans retinoic acid. **A** shows expression of phospho-extracellular signal-regulated kinase (phosphor-ERK1/2) protein in human scleral fibroblasts (HSFs) visualized with indirect immunofluorescence. The nuclei were stained with propidium iodide dye (red: **A**1, **B**1, **C**1) and the primary antibody was labeled with daylight 488-conjugated secondary antibody (green: **A**2, **B**2, **C**2). Panels **A**1-**A**3 shows a negative control incubated in 0.01 M phosphate buffered saline (PBS) with no primary antibody. Panels **B**1-**B**3 shows cells incubated in control medium. Phospho-p44/42 protein is seen expressed in the cytoplasma and nuclei, and the cells display many protusions. Panels **C**1-**C**3 shows cells incubated with all-trans retinoic acid (ATRA) for 1 h. As can be seen, the expression of phospho-ERK 1/2 is decreased and the morphology of the cells changed. The original magnification was 400 X, and the scale bar=20 µm. Panel **B** shows western blot for phospho-ERK1/2, ERK1/2, and antiglyceraldehyde-3-phosphate dehydrogenase (GAPDH). There is significant downregulation of phosphor-ERK 1/2 protein after incubation with 1 µmol/l ATRA from 30 min to 24 h, but no change of ERK 1/2 protein. As can be seen the bar graph of panel **C**, expression of phospho-ERK 1/2 protein relative to GAPDH density in cells incubated with ATRA is significantly and increasingly depressed from 30 min to 24 h (n=3, *p<0.05, **p<0.01). In contrast, as seen in the bar graph of panel **D**, incubation with ATRA did not change expression of ERK 1/2 protein relative to GAPDH density (n=3).

The HSFs were treated with 1 μmol/l ATRA for 10 min, 30 min, 1 h, 8 h, or 24 h. After treatment with 1 μmol/l ATRA for 1 h, the protein expression of phospho-ERK 1/2 had decreased; the HSFs cells appeared smaller, and displayed fewer cell protrusions than in the control group (Figure 2A). Western blot analysis verified the results, showing downregulation of the phospho-ERK 1/2 protein in cultures exposed to 1 μmol/l ATRA for 30 min (1.00 versus 0.71, p=0.01), and substantial downregulation after exposure for 1 h (1.00 versus 0.59, p<0.01), 8 h (1.00 versus 0.54, p<0.01), and 24 h (1.00 versus 0.52, p<0.01; Figure 2B,C). There were no significant changes in the total ERK protein (phosphorylated and non-phosphorylated) in HSFs treated with ATRA (Figure 2B,D).

### Increased phosphorylation of c-Jun N-terminal kinase in human scleral fibroblasts exposed to All-trans retinoic acid

**Figure 3:**
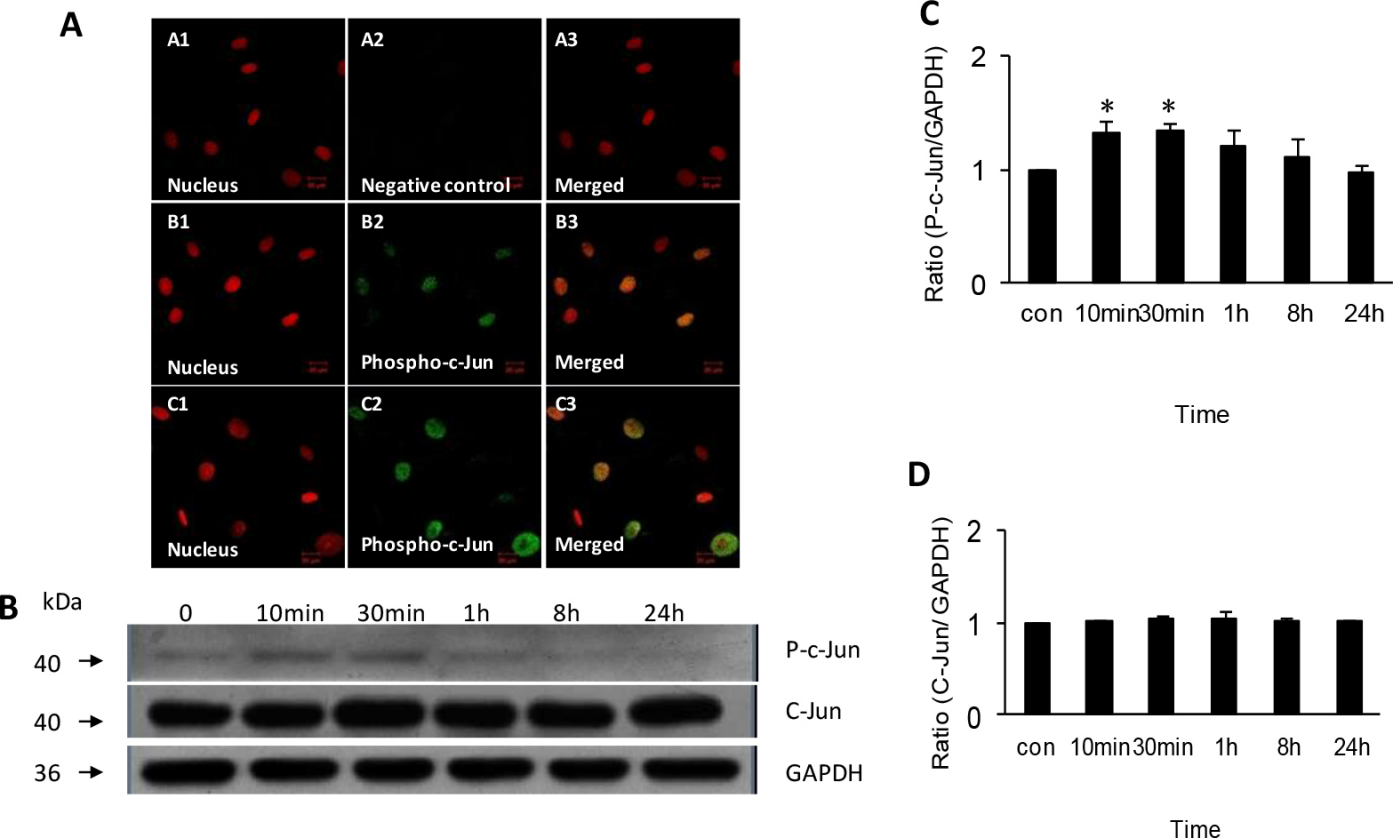
Expression and activation of phospho-c-Jun and c-Jun protein in human scleral fibroblasts treated with 1 µmol/l all-trans retinoic acid. Panel **A** shows expression of phospho-c-Jun protein in HSFs visualized with indirect immunofluorescence. The nuclei were stained with propidium iodide dye (red: **A**1, **B**1, **C**1) and the primary antibody was labeled with daylight 488-conjugated secondary antibody (green: **A**2, **B**2, **C**2). **A**1-**A**3 shows a negative control incubated in 0.01 M phosphate buffered saline (PBS) with no primary antibody. **B**1-**B**3 shows cells incubated in control medium. **C**1-**C**3 shows upregulation of phospho-c-Jun protein expression in cells incubated with all-trans retinoic acid (ATRA) for 1 h. The original magnification was 400 X, and the scale bar=20 µm. Panel **B** shows western blot for phospho-c-Jun, c-Jun, and antiglyceraldehyde-3-phosphate dehydrogenase (GAPDH). There is significant upregulation of phospho-c-Jun protein after incubation with 1 µmol/l ATRA from 10 min to 30 min, but no change of c-Jun protein. The bar graph in figure 3C shows significant and increasing upregulation of phospho-c-Jun protein expression relative to GAPDH density in cells incubated with ATRA from 10 min to 30 min (n=3, *p<0.05). In contrast, as seen in the bar graph of panel **D**, incubation with ATRA did not change expression of c-Jun protein relative to GAPDH density (n=3).

Indirect immunofluorescence showed that the protein of phospho-JNK was expressed and distributed only in the nucleus of HSFs. After treatment with 1 μmol/l ATRA for 10 min, the expression of phospho-JNK had increased (Figure 3A). Western blot showed that the phospho-JNK protein was significantly upregulated by exposure to 1 μmol/l ATRA for 10 min (1.00 versus 1.33, p<0.01) and 30 min (1.00 versus 1.34, p<0.01), and then returned to preexposure levels (Figure 3B,C). There was no significant change in the total JNK protein in response to treatment with ATRA (Figure 3B,D).

### No activation of phospho-p38 in human scleral fibroblasts exposed to all-trans retinoic acid

**Figure 4:**
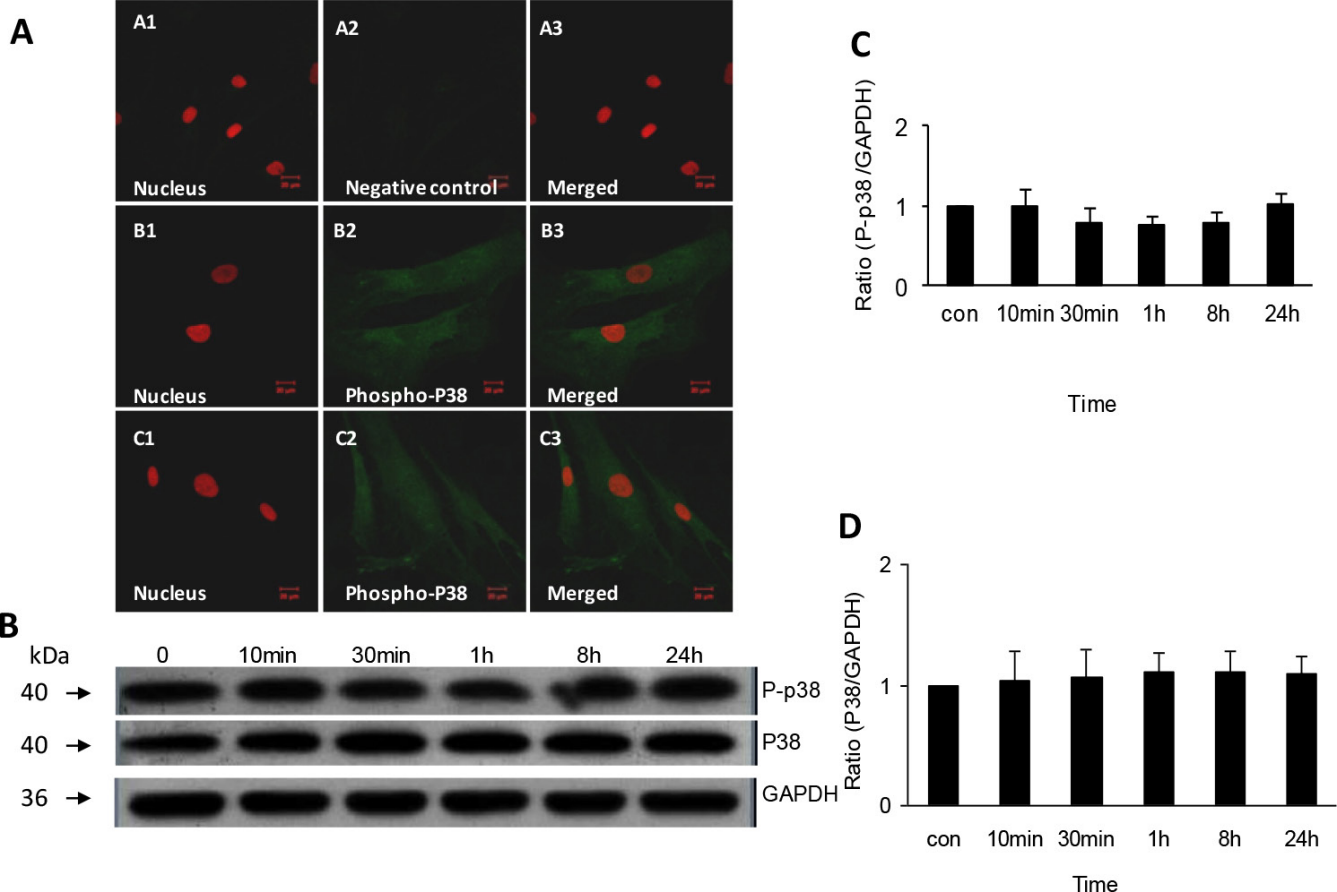
Expression and non-activation of phospho-p38 and p38 protein in human scleral fibroblasts treated with 1µmol/L all-trans retinoic acid. Panel **A** shows expression of phospho-p38 protein in HSFs visualized with indirect immunofluorescence. The nuclei were stained with propidium iodide dye (red: **A**1, **B**1, **C**1) and the primary antibody was labeled with daylight 488-conjugated secondary antibody (green: **A**2, **B**2, **C**2). Panels **A**1-**A**3 shows a negative control incubated in 0.01 M phosphate buffered saline (PBS) with no primary antibody. Panels **B**1-**B**3 shows cells incubated in control medium. Phospho-p38 protein is expressed in the cytoplasm and nuclei. Panels **C**1-**C**3 shows that there is no change in the expression of phospho-p38 protein in cells incubated with all-trans retinoic acid (ATRA) for 1 h. The original magnification was 400 X and the scale bar=20 µm. Panel **B** shows western blot for phospho-p38, p38, and antiglyceraldehyde-3-phosphate dehydrogenase (GAPDH). There is no change of phospho-p38 or p38 protein after incubation with 1 µmol/l ATRA. The bar graph in panel **C** shows that there is no effect of ATRA on expression of phospho-p38 protein relative to GAPDH density (n=3). The bar graph in panel **D** shows that there is no effect of ATRA on expression of p38 protein relative to GAPDH density (n=3).

Indirect immunofluorescence showed that the protein of phospho-p38 was expressed and distributed in the HSFs’ cytoplasm and nucleus. After treatment with 1 μmol/l ATRA for 1 h, no change in the expression of phospho-p38 was apparent. Western blot showed downregulation of phospho-p38 from 30 min to 8 h after exposure to ATRA, but the differences were not statistically significant (1.00 versus 0.79, p=1.00). Compared to the control group, the protein of p38 showed no significant change from 10 min to 8 h after exposure to ATRA (Figure 4).

### Retinoic acid antagonist LE135 inhibiting the activation of extracellular signal-regulated kinase and c-Jun

**Figure 5:**
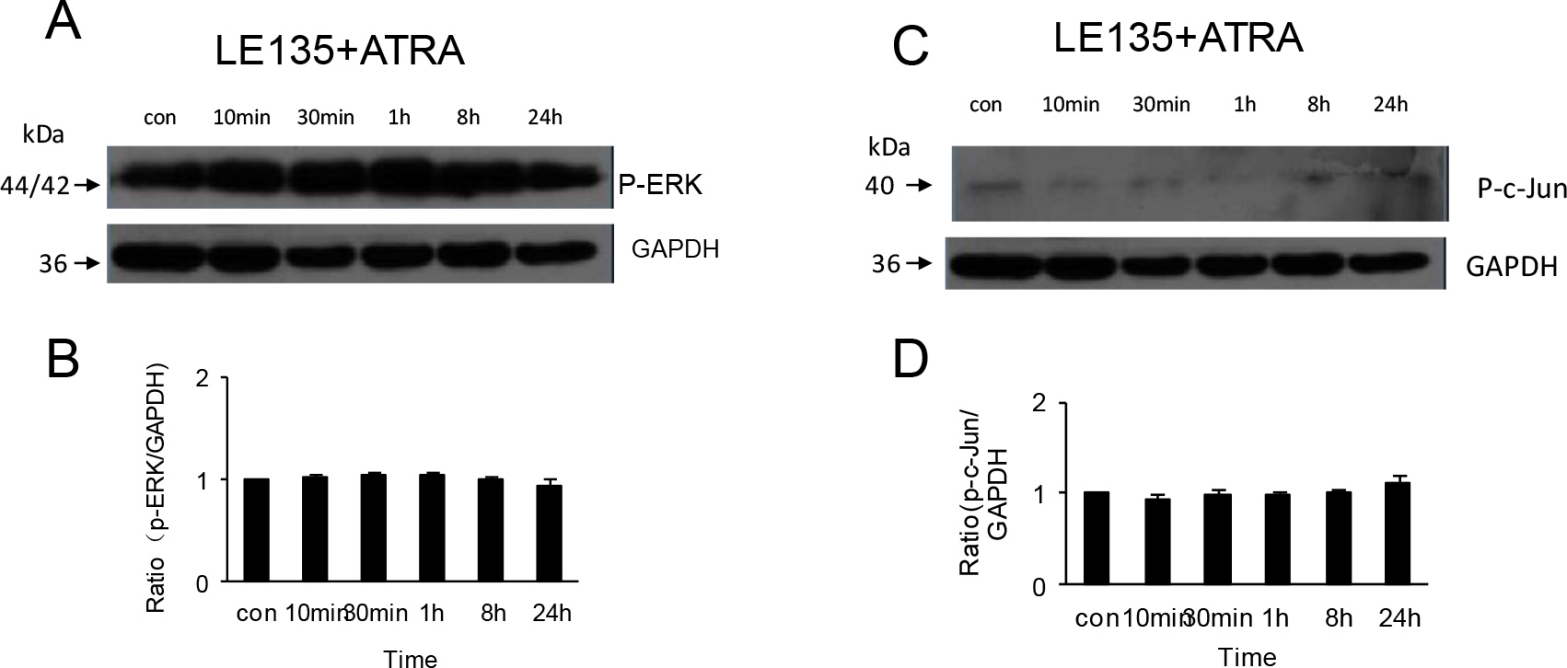
Activation of phospho-ERK1/2 and phospho-c-Jun protein in human scleral fibroblasts treated with 1 µmol/l all-trans retinoic acid is inhibited by pre-treatment with 1 µmol/l LE135. As the western blot of panel **A** shows, incubation with ATRA did not change the expression of phospho-ERK1/2 protein in HSFs pretreated with LE135. The bar graph of panel **B** shows that the expression of phospho-ERK1/2 protein relative to GAPDH density in cells incubated with ATRA is unchanged after pretreatment with LE135 (n=3). As shown in the western blot of panel **C**, the expression of phospho-c-Jun protein incubated with ATRA is unchanged in HSFs pretreated with LE135. The bar graph of panel **D** shows that the expression of phospho-c-Jun protein relative to GAPDH density in cells incubated with ATRA is unchanged after pretreatment with LE135 (n=3).

To study whether the antagonist of RARβ, LE135, inhibited the response of ERK 1/2 and JNK to ATRA exposure, HSFs were pretreated with 1 μmol/l LE135 for 24 h and then treated with 1 μmol/l ATRA for 10 min, 30 min, 1 h, 8 h, or 24 h. Western blot showed that there were no significant changes in phospho-ERK 1/2 (p=1.00) and phospho-c-Jun (p>0.5) protein expression in HSFs after exposure to ATRA (Figure 5).

## Discussion

Our results confirm earlier findings of an inhibitory effect of retinoic acid on the proliferation of HSFs [10]. We also found that treatment with ATRA changes the morphology of HSFs. In an earlier study, we found that ATRA enhances RARβ mRNA and protein expression (our unpublished data). In the present study, we investigated whether the activation of the MAPK signal pathway, which includes ERK 1/2, JNK, and p38, is modulated by ATRA. Our results suggest that ATRA persistently decreases the phosphorylation of ERK 1/2 MAPK and transiently increases the phosphorylation of JNK MAPK, whereas no change was detected in p38 MAPK over the 24 h study period. These ATRA-induced changes in activation of ERK 1/2 and JNK MAPK were abolished by pretreatment with the RARβ antagonist LE135.

Retinoic acid (RA) appears to play a role in controlling proliferation and differentiation in various types of cells. Thus, ATRA has been shown to inhibit the proliferation of retinal pigment epithelium [19] and lacrimal acinar cells [20]. As a potential treatment agent, RA exerts antitumorigenic effects in many types of cancers, such as Kaposi's sarcoma, bladder cancer, ovarian carcinoma, head and neck squamous cell carcinoma, neuroblastoma, and breast cancer [21,22]. RA also shows antiangiogenic effects in several systems, inhibiting proliferation in vascular, smooth muscle cells. RA also decreases inflammation in rheumatoid arthritis [21]. We have found that ATRA can inhibit proliferation of HSFs cultured in vitro. The number of live HSFs decreased and the morphology of HSFs changed after incubation with ATRA. The result of the current study is consistent with our previous study [10]. The MAPK pathway is known to regulate cell proliferation, differentiation, and apoptosis [11,23]. Earlier studies indicated that RA inhibits cell proliferation by suppressing phosphorylation of ERK 1/2 [22,24-26]. Our findings are in accordance with these results, as we also found reduced phosphorylation of ERK 1/2 in HSFs exposed to ATRA.

In addition to the effect on the ERK, RA modulates the activation of JNK [27] and p38 [28-30]. Phosphorylation of the DNA binding protein JNK increases the transcriptional activity. JNK is a component of the AP-1 transcription complex, which is activated in response to environmental stress, radiation, and growth factors [11]. In contrast to our findings for ERK 1/2, increased phosphorylation of JNK in HSFs exposed to ATRA does not seem correspond to the observed suppression of HSF cell proliferation. This could indicate that cell proliferation is more sensitive to changes in activation of ERK 1/2 than to changes in activation of JNK. In diabetic rat liver cells, DNA damage is differentially correlated with the activation of ERK 1/2 and JNK. Thus, significant DNA damage is accompanied by increased activation of JNK and lower DNA damage with increased activation of ERK [31].

Although the p38 MAPK pathway may be activated by ATRA in other cells [28-30], the current study shows no change in phospho-p38. The results are similar to the former studies, which indicate that ATRA can activate one or two classical MAPK pathways, with little or no change in the other pathway [32]. This reveals that which MAPK pathway plays a role in cell proliferation probably depends on different stimuli or cell types.

Our finding that pretreatment with LE135, a selective antagonist of RARβ, abolishes the change in ERK 1/2 and JNK MAPK activation after exposure of the HSFs to ATRA suggests that the effects of ATRA are mediated by RARβ. We previously found that the expression of RARβ in HSFs, at the mRNA and protein level, is enhanced after exposure to ATRA. The maximum inhibiting effect of LE135 on the ATRA-induced transcriptional activation of RARβ is obtained at 1 μmol/l [13], which is the same concentration as in our experiment and other recent studies [16,17]. Guinea pigs with experimental myopia display elevated levels of RA and RARβ in the retina, and there appears to be positive feedback between increased RA and elevated RARβ [33]. In marmosets that developed elongated eyes in response to visual deprivation, elevated levels of RA correlated with decreased synthesis of glycosaminoglycans [34]. The decreased synthesis of extracellular components such as glycosaminoglycans in sclera from mammals developing eye elongation could hypothetically be caused by the number of scleral fibroblasts being reduced in response to elevated levels of RA. However, in vivo and in vitro studies have shown that exposure to ATRA also produces more specific effects in scleral fibroblasts. Thus, ATRA increases expression of fibulin-1, an important connective tissue extracellular matrix protein and a ligand for aggrecan, and at the same time decreases expression of aggrecan, a glycosaminoglycan-containing proteoglycan that plays an important role in sclera remodeling, in guinea pig sclera and HSFs. The inverse relationship between fibulin-1 and aggrecan seems to depend on a chain effect in which increased level of fibulin-1 enhances enzymatic degradation of aggrecan. Our previous study showed that fibulin-1 not only is expressed in human sclera but also can be induced by ATRA, a molecule known to be involved in regulating eye growth [10].

In summary, our data suggest that ATRA inhibited HSF proliferation by a mechanism associated with modulation of ERK 1/2 and JNK activation and depends on stimulation of RARβ. MAPKs need to translocate from the cytoplasm into the nuclei to regulate transcription factors, such as c-Jun, in the nuclei [35] and participate in regulating cell proliferation and changes in morphology. This study may provide clues to the mechanism of ATRA that inhibits the proliferation of HSFs in vitro.
